# Authorship and Citizen Science: Seven Heuristic Rules

**DOI:** 10.1007/s11948-024-00516-x

**Published:** 2024-10-29

**Authors:** Per Sandin, Patrik Baard, William Bülow, Gert Helgesson

**Affiliations:** 1 Department of Crop Production Ecology, Swedish University of Agricultural Sciences, Box 7043, 75651 Uppsala, Sweden; 2 Present Address: Department of Applied Animal Science and Welfare, Swedish University of Agricultural Sciences, Uppsala, Sweden; 3 Centre for Research Ethics & Bioethics (CRB), Uppsala University, Uppsala, Sweden; 4 Department of Learning, Informatics, Management and Ethics, Karolinska Institutet, Stockholm, Sweden

**Keywords:** Citizen science, Authorship, Credit, Vancouver guidelines, International Committee of Medical Journal Editors (ICMJE)

## Abstract

Citizen science (CS) is an umbrella term for research with a significant amount of contributions from volunteers. Those volunteers can occupy a hybrid role, being both ‘researcher’ and ‘subject’ at the same time. This has repercussions for questions about responsibility and credit, e.g. pertaining to the issue of authorship. In this paper, we first review some existing guidelines for authorship and their applicability to CS. Second, we assess the claim that the guidelines from the International Committee of Medical Journal Editors (ICMJE), known as ‘the Vancouver guidelines’, may lead to exclusion of deserving citizen scientists as authors. We maintain that the idea of including citizen scientists as authors is supported by at least two arguments: transparency and fairness. Third, we argue that it might be plausible to include groups as authors in CS. Fourth and finally, we offer a heuristic list of seven recommendations to be considered when deciding about whom to include as an author of a CS publication.

## Introduction

Citizen science (CS) is an umbrella term for research where the research process and its outcomes are dependent on contributions from volunteers. It comes in many shapes.^1^ One common form is represented by projects in biology or ecology where volunteer participants report their observations of various species of e.g. birds, insects or plants, which are then stored in databases and used for research. Examples are projects hosted by the Swedish Species Observation System (Artdatabanken, 2024). In another field, GalaxyZoo (Zooniverse, 2023) allows volunteers to categorize galaxies and astronomical objects in photographs online. The ‘Polymath project’ is a blog started 2009 by Fields medal winner Timothy Gowers that allows volunteers to participate in discussing and solving mathematical problems (Gowers & Nielsen, 2009), thereby contributing intellectually rather than through mere data collection. In the Eterna game project (Eterna, 2023), volunteers suggest RNA sequence designs and vote which possible solutions are to be subsequently tested in laboratories by researchers at Stanford University. Here the CS contribution consists in conception and design. Thus, volunteers’ contributions are manifold and substantial.

Sometimes those contributions are formally recognized and credited. An astronomical phenomenon known as ‘Hanny’s Voorwerp’ is named after its GalaxyZoo volunteer discoverer Hanny van Arkel who was recognized as a co-author of several academic publications on the topic (e.g. Lintott et al., 2009). In some cases, ‘EteRNA participants’ appear as *a* co-author, along with appendices of numerous online users involved in the work (Koodli et al., 2019; Lee et al., 2014). The Polymath project has generated some articles that have been published under the collective pseudonym ‘D. H. J. Polymath’ in order to recognize volunteer contributions (e.g. Polymath, 2014). (The ‘initials’ are presumably derived from topics of some of the first papers: density Hales-Jewett numbers, Tao, 2010.) Judging by the number of CS projects and the relatively few cases where authorship credits of CS participants are provided, these examples are exceptions rather than the rule—volunteer contributions to CS are often not credited (Sandin & Baard, 2024; Dickinson et al., 2012; Ward-Fear et al., 2020).

In this article, we focus on authorship in CS. We assess to what extent citizen science provides a challenge to established authorship guidelines, such as the guidelines from the International Committee of Medical Journal Editors (ICMJE), known as the ‘Vancouver guidelines’ (ICMJE, 2023). In particular, we discuss whether those guidelines should be maintained in their present form in the case of CS or whether they ought to be changed or amended. We acknowledge that CS contributors should be given recognition for their contributions (Resnik, 2019b; Resnik et al., 2015; Ward-Fear et al., 2020; Riesch & Potter, 2014; Tauginienė, 2019) while arguing that existing authorship guidelines in many cases permit, or even mandate, exclusion of CS contributors from authorship. We discuss the reasonableness of this. Further, we discuss the need of certain modifications of existing authorship guidelines in order to better account for group contributions to CS.

The paper is structured as follows: First we review existing authorship guidelines and their applicability to CS. Second, we assess the claim that existing authorship guidelines, and the ICMJE’s Vancouver guidelines in particular, can lead to exclusion of citizen scientists whose contributions should be recognized through authorship. We find the idea of including citizen scientists as authors plausible and supported by at least two arguments: transparency and fairness. Third, we argue that it might be plausible to include groups as authors in CS. Finally, we offer a heuristic list of seven recommendations to be considered when deciding about whom to include as an author of a CS publication.

## Academic Authorship and the ICMJE Recommendations

To determine when and under what circumstances those who make contributions as citizen scientists should be included as authors on research papers, we first need to consider academic authorship as such. The question of how to allocate authorship on co-researched papers is an important one. In contemporary academia, individual researchers as well as research institutions are typically evaluated on the basis of their publications, for instance, when researchers apply for positions or grants or when departments are up for review. The idea behind this is that authorship tracks certain achievements in the research process, and, by extension, desirable skills in the researchers who are listed as authors. The prevalent idea of academic authorship is therefore *desert-based*, meaning that authorship should be awarded to someone because they *deserve* it, having earned it through their contributions. Furthermore, authorship implies responsibility: Authors of a work are expected to be accountable for its content (Helgesson et al., 2018; Helgesson & Eriksson, 2018; Hansson, 2017).

There is no real consensus as to what makes someone deserving of authorship, or of a more or less prominent position in the author list (Marušic et al., 2011; Bozeman & Youtie, 2016; Patience et al., 2019; Helgesson & Eriksson, 2019). As an attempt to bring order to this matter, a number of guidelines have been proposed and adopted (Hansson, 2017; Osborne & Holland, 2009; Shamoo & Resnik, 2009). These guidelines can be seen as normative codifications of the view that authorship includes both credit and responsibility. The most well-known example is the ICMJE Recommendations (‘the Vancouver guidelines’), developed by the International Committee of Medical Journal Editors (ICMJE, 2023). They recommend that authorship be based on the following four criteria:

1. Substantial contributions to the conception or design of the work; or the acquisition, analysis, or interpretation of data for the work; AND
2. Drafting the work or reviewing it critically for important intellectual content; AND
3. Final approval of the version to be published; AND
4. Agreement to be accountable for all aspects of the work in ensuring that questions related to the accuracy or integrity of any part of the work are appropriately investigated and resolved. (ICMJE, 2023).

The ICMJE recommendations are meant to provide action-guidance and ensure some degree of uniformity concerning the allocation of authorship. Such uniformity is important not least in order to ensure fair competition among researchers, which presuppose that everyone plays by the same rules. Even though they are not legally binding, the ICMJE recommendations are widely accepted in the scientific community and have been adopted as in-house policy by a number of higher education institutions, journals and publishing houses (see e.g. ALLEA, 2023; Springer, 2023). Most, if not all researchers, can therefore be expected to follow these recommendations. That said, there are discrepancies between the guidelines and actual practice (Helgesson and Eriksson, 2018; Bülow and Helgesson, 2018; Logan et al., 2017), and the ICMJE are not without critics. Among other things, it has been argued that the guidelines are too vague (Helgesson & Eriksson, 2018; Bülow & Helgesson, 2018; Cutas & Shaw, 2015) and overly restrictive (Shaw, 2011; Mofatt, 2018). Another criticism is that they, despite the attempt to ensure uniformity, fail to recognize and respect the variety of distinct accounts of authorship that prevail in different academic disciplines (Moffatt, 2018). One example here is CS, which involves roles and tasks that are not credited in the current system but, some would argue, should be credited through authorship under some conditions (Resnik, 2019b; Resnik et al., 2015; Ward-Fear et al., 2020; Riesch & Potter, 2014; Tauginiené, 2019). In what follows, we will take a closer look at some of the arguments underlying this type of criticism.

## Authorship and Citizen Science

### Do the ICMJE Authorship Criteria Unfairly Exclude CS Contributors?

Distilling the criticism directed by the CS community at current authorship guidelines, we can identify the following premise:

- Existing principles to guide authorship, and the ICMJE guidelines in particular, may lead to exclusion of citizen scientists whose contributions should be recognized through authorship.

This notion is supported by several considerations.

#### Necessary and Substantial Contributions

Resnik and colleagues note that the collective contribution from volunteers is non-negligible and in many projects amounts to substantial hours of labour (Resnik et al., 2015, p. 476). In fact, one rationale for running CS projects at all is that they make it possible to address research questions ‘at scales that would be unachievable through professional science alone’, for instance the collection of continental-wide datasets in ecology (Miller-Rushing et al., 2012, p. 286). Hence, one argument making a case that volunteers should be credited, with authorship or otherwise, states that the contribution is *necessary* for the success of the research project. The extent of contributions will differ. One could make the claim that even organisational chores are necessary for conducting research and output, but this would be highly questionable to merit credit in the form of authorship, with the main argument that the contributions should be specific to the project at hand [see however Liboiron et al. (2017)]. In contrast, gathering and analyzing data, participating in discussions on hypotheses, and similar activities, are more consistent with conventional views on contributions. While many participants in CS projects primarily help gather and collect data, volunteers can also play central roles in formulating the study aims and research design and instigation of community-based research projects (Resnik et al., 2015). We argue that some such CS contributions deserve recognition through authorship; still, they are often neglected (Cooper et al., 2014; Ward-Fear et al., 2020). This criticism is not unique to CS—similar concerns about unfair exclusion or downgrading of authors occur in other kinds of research as well, among them participatory research (Liboiron et al., 2017; Sarna-Wojcicki et al., 2017).

#### Unfair Guidelines or Unfair Practices?

Another concern is that current practices and existing guidelines, such as the ICMJE recommendations, are unfair and exclude deserving authors. For instance, Ward-Fear et al. mention ‘the breadth of situations in which unintentional *discrimination* occurs as a result of rigid authorship protocols’ (Ward-Fear et al., 2020, p. 187, emphasis added). This position relies on the same desert-based idea of authorship as the ICMJE recommendations codify. However, unfair exclusion of deserving contributors can be the fault either of unfair *guidelines* or unfair *practices* among researchers running CS projects. If researchers do not comply with the guidelines, the guidelines themselves might not be to blame for the outcome.

It may thus well be the case that the problem lies in non-compliance rather than with the guidelines. Resnik et al. (2015) claim that CS volunteers often *have* made important contributions to a certain research project. If they have, this suggests that they probably made a substantial contribution of the sort dictated by the ICMJE recommendations. In such cases they should be provided with the opportunity to fulfil also the other criteria for authorship, as the Vancouver guidelines themselves unambiguously prescribe: ‘all individuals who meet the first criterion should have the opportunity to participate in the review, drafting, and final approval of the manuscript’ (ICMJEn, 2023; Resnik et al., 2015). If they are not given this opportunity, it is a breach of the guidelines.

It is in fact a feature of several CS projects that a small number of ‘superusers’ contribute most of the material (Macphail et al., 2020). A perhaps extreme example in this regard is the online project kbhbilleder.dk. In this project, volunteers tag historical images from Copenhagen. Among a total of 2433 participants, there were seven users who accounted for about 94% of the work (Hansson & Dahlgren, 2022). In such cases, the ‘superusers’ can definitely be said to have made a substantial scientific contribution, and it would be entirely in line with the guidelines to give those ‘superusers’ the opportunity to fulfil all the criteria for authorship.

#### Strategic Considerations

In addition to purely desert-based arguments, it is sometimes pointed out that adopting more inclusive criteria for authorship would benefit science, and that not including citizen scientists as authors ‘is unwise if researchers hope to maintain credibility and collaborate with groups [of volunteers] in the future’ (Ward-Fear et al., 2020). There is some reason to believe that formal recognition is a motivational factor for some citizen scientists (Ganzevoort et al., 2017; Tauginienė et al., 2021, p. 405; Rotman et al., 2012). If motivation for participation is dependent on receiving recognition, then lack of recognition may lead to fewer people being motivated to participate, with an obvious potential cost to research. However, few participants seem to regard authorship as the *only* form of recognition. Other forms (acknowledgment, being mentioned in the contributor list, etc.) may thus be sufficient and granting authorship would then not be necessary.^2^ Besides, opening up for exceptions from strictly following the criteria would probably make it even more difficult to battle the overinclusion that takes place within many research fields today and where it might be more of a concern than it is in CS.

## Groups as Authors

The discussion so far suggests that individual volunteers should be credited for their contributions. If they have made a substantial enough contribution (the first ICMJE criterion for authorship), they should be given the opportunity to also fulfil the other criteria of the recommendations (involvement in drafting or reviewing the paper, approval of final version, and accountability). If they actually do fulfil all criteria, they should be granted authorship. A complication, however, is the following: The contributions of volunteers may be collectively important, yet insufficient *individually* to fulfil the ICMJE criterion of substantial contribution, although their *joint* efforts *very much* amount to a ‘substantial contribution’ to the paper. In such situations, ICMJE recommends that everyone contributing should be recognized for their efforts in the acknowledgments. This might suggest that groups could be listed as authors *qua* groups (Resnik et al., 2015; Ward-Fear et al., 2020).

It is not obvious that the Vancouver guidelines are consistent with the suggestion that authorship proper could be awarded to groups. For now, we leave individual volunteers fulfilling authorship criteria aside—they should be treated as any other substantially contributing researcher—in order to fully focus on group authorship. This is covered in the guidelines as follows: ‘When submitting a manuscript authored by a group, the corresponding author should specify the group name if one exists, and clearly identify the group members who can take credit and responsibility for the work as authors.’ (ICMJE, 2023). The intentions are not entirely clear from this quote, but on the perhaps most reasonable interpretation, the guidelines are not open to genuinely attributing authorship to groups. A group may be mentioned, but only individuals are proper authors. Another possible interpretation is that the group may also be an author. In that case, if an individual is a member of a group to which authorship is attributed, this does not mean that he or she can count this as ‘full’ individual authorship. For individuals to be given full authorship credit, they have to fulfil the authorship criteria as individuals.

### Groups Making Substantial Contributions: Transparency and Fairness

The question of group authorship is philosophically interesting. As we have seen, a group’s collective effort can be such that it reaches above the threshold of a substantial contribution, even though none of the individual contributions is so substantial (Resnik, 2019b, p. 4). There are reasons for taking the issue of group credit and authorship seriously. One reason is that the omission of a public accreditation of important contributions makes the work incompletely described and easily gives the misleading impression that the professional researchers in the project made a larger contribution to the work than they in fact did (Resnik et al., 2015, p. 480). Hence, there is a *transparency* argument in favor of giving credit to volunteer contributions that played a significant role in the project. However, this argument primarily stresses the need to adequately describe who contributed with what—it is not obvious that transparency is lacking if nothing is said about the group deserving authorship, if individual contributions, and what their work adds up to, are well described.

Apart from the transparency argument, there is a *fairness* argument. The ICMJE Recommendations stipulate that individual researchers who fulfil the ‘substantial contribution’ criterion deserve to be given opportunity to fulfil the other authorship criteria as well. Thus, it would be unfair not to grant groups who are similarly deserving the same opportunity.

However, according to the ICMJE Recommendations, making a substantial contribution to research is a necessary but not a sufficient criterion for authorship. The other three criteria need to be fulfilled as well. An argument against this position, heard long before discussions of group authorship, is that it should be enough to make a substantial contribution to qualify as author. However, the other points are needed to tie authorship not only to scientific credit but to responsibility for the research as well. We find the idea of responsibility important. It seems unreasonable to get credit for something one is not willing to assume responsibility for. So it needs to be discussed whether groups, *qua* groups, are not only able to make a substantial contribution, but also to fulfil the other authorship criteria. If not, it needs to be considered whether or not this, after all, points to a need to revise the ICMJE criteria to accommodate group authorship.

Substantial contribution is a central ‘gatekeeping’ condition for the other three authorship conditions, since ‘all individuals who meet the first criterion should have the opportunity to participate in the review, drafting, and final approval of the manuscript’ (ICMJE, 2023). However, even if we accept that groups can fulfil the first ICMJE criterion (substantial contribution), complications remain regarding the other three.

### Critical Review and Final Approval

Regarding the second criterion—drafting or critically reviewing the paper for important intellectual content—nothing excludes that citizen scientists participate in revising the manuscript for important intellectual content and then approve of the final version of the manuscript to be submitted to a journal, hence providing revision and approval from the group. Still, it is debatable both how much needs to be delivered from the group for the criteria to be fulfilled and how much is reasonable to ask. Normally, the second criterion is thought to have two functions: that collaborators read critically to spot errors and other needs for revision and that they recognize what they assume responsibility for. However, as discussed in the next section, it is debatable what level of responsibility citizen scientists need to assume. It will be the task of the lead author(s) to exercise their judgment.

What conditions to raise for groups as co-authors regarding approval of the final manuscript version is not obvious. One view could be that everyone in the group involved with the work on the paper should approve the last version. In the case of numerous strong-willed contributors, such a condition might risk blocking many papers from ever getting submitted. This third criterion of author-approval of the submittable manuscript version is tied to ideas of responsibility. If the entire CS group involved in the work unanimously refuses to accept the final version, it should not be submitted in that form with the group as co-author. However, there needs to be some procedure in place to avoid or resolve situations of *hostage authorship*. Hostage authorship occurs when ‘the researchers of an article cannot proceed and finish the article unless the conditions raised by [an undeserving contributor] are fulfilled’ (Bülow & Helgesson, 2018). Examples include when clinicians in medicine refuse to provide data unless they are awarded authorship, even though they do not fulfil the rest of the criteria of the Vancouver Guidelines.

### Groups and Accountability

Group authorship also raises important questions with respect to accountability. Suppose a group containing 2,000 birdwatchers is listed collectively as co-author of a paper and there are some questions regarding the integrity of some part of the work—perhaps it turns out that some birdwatchers have deliberately misreported their observations. Is the whole group to be held accountable (perhaps banned from contributing in the future)? Would it depend on whether the group was structured in some particular way (French, 1995)?

As has been discussed elsewhere in moral philosophy, it is conceivable that a large group is collectively responsible for some harm having occurred even if the contribution from each individual is marginal (Parfit, 1984). In such instances, it seems mistaken both to absolve the whole collective from responsibility and to ascribe responsibility to a single individual. The same considerations apply to benefits. We thus follow Resnik here in viewing responsibility and credit [e.g. authorship] ‘as two sides of the same coin’ (Resnik, 1998, p. 62).

However, there are reasons to resist the conclusion that all co-authors are equally accountable for all parts of a research article (see for instance Helgesson & Eriksson, 2018, Helgesson et al., 2018). This holds also for ‘traditional’ co-authored articles, where all authors are professional researchers and there are no citizen scientists involved. Some co-authors may in general ‘lack some of the competences needed to assess all aspects of the paper’ (Hansson, 2017, pp. 100–101) and can therefore not assume responsibility to the same extent as those with all, or more, relevant competences. We have already underlined the great variety in CS projects. It is likely that most participants in ‘top-down’ projects who carry out some practical task (like collecting water samples) will not be able to engage in critically reviewing a manuscript for important intellectual content. One may even go as far as to think that this might, for some research, be scientifically pointless for most volunteers.

There are two options for getting out of this quandary: Either (a) one insists that accountability is an essential aspect of authorship, so someone who cannot be held accountable cannot be an author, or (b) one defends a view on authorship that requires less, such as focusing on having made a substantial contribution to the work. The former is the approach taken by the ICMJE. It is also the current consensus in the publishing industry redgarding authorship for generative AI tools, such as Chat GPT. Since such tools lack accountability, they should not be listed as author of a paper (Kaebnick et al., 2023, p. 1).^3^

The latter approach would mean that citizen scientists would still have to shoulder the responsibility for their own contributions, but there would be no general requirement beyond that. This position could be supplemented by a thorough and systematic use of contributorship descriptions (e.g. Whetstone & Moulaison-Sandy, 2020). That way credit is given where it is due.

The focus of the present paper has been on unfair exclusions of deserving contributors. Before wrapping up, we should note, however, that there may well be situations were volunteers who have made significant contributions may wish not to be listed as co-authors, for good reasons.^4^

## Seven Heuristic Rules

Above, we have argued that considerations of desert, transparency and fairness speak in favor of including CS participants as authors, and that this is compatible with the Vancouver guidelines. Thus, we do not propose radical reform of existing practices for authorship. It is possible, however, that future developments might require such reform, for example in relation to group contributions.

In light of the above considerations, we propose the following heuristic list of recommendations about whom to include as an author of a CS publication:

- 1. As far as possible, respect existing guidelines (such as ICMJE’s Vancouver guidelines).

A related point is perhaps obvious:

- 2. Ensure that no authors who fulfil the criteria of the Vancouver guidelines are left out.

As we noted above, some contributors might be unfairly excluded not because there is anything in existing guidelines that excludes them, but because of non-compliance with those guidelines. This should be checked first. In cases where guidelines do seem to exclude CS participants as authors, there are a number of additional possibilities to explore before deciding that the CS participants do not qualify.

- 3. Use existing resources for specifying roles of contributors.

In case the journal or publisher utilizes some conventions for clarifying the various roles of contributors, one should make use of them. One example is Contributor Role Taxonomy (CRediT) (Allen et al., 2019; Whetstone & Moulaison-Sandy, 2020). Other resources propose some sort of explicit and quantitative assessment of author contributions, such as Clement’s (2014) authorship matrix, and the ‘scorecard’ from the American Psychological Association (n.d.), which in turn builds on Winston (1985) and previous work by the APA Ethics Committee.

- 4. Look to nearby fields.

It might be advisable to consider practices from some nearby discipline or academic field to see what contributions tend to be seen as sufficient for authorship. One may, for instance, in certain circumstances find it reasonable to particularly stress substantial scientific contributions and responsibility acceptance, as suggested by Hansson (2017), while putting a lesser emphasis on critical revision of the manuscript. Even in medicine, where the Vancouver guidelines are often strictly applied, collaborations between microbiologists in the lab and physicians at the hospital clinic often find it acceptable that clinicians may not always be competent to provide constructive criticism to much of the content of the papers, then restricting their review to parts they have contributed to and are familiar with. Hansson’s proposal might be a bottom line, so that if a CS contributor’s scientific contribution is *very* strong, this may speak in favour of including the contributor as an author—even if all four criteria of the Vancouver guidelines are not fulfilled.

- 5. Apply a wide conception of contributions.

The Vancouver guidelines’ conception of ‘substantial contributions’, explicitly relating to authorship, are contributions ‘to conception or design of the work; or the acquisition, analysis, or interpretation of data for the work’ (ICMJE, 2023). When discussing contributions more broadly, for a contributor list, it is obvious that there are many other contributions that may play a significant role for the success of a research project. This holds true for both CS and ‘conventional’ non-CS science. The Civic Laboratory for Environmental Action Research (CLEAR) provide examples of ‘care work’ such as ‘training new members on protocols; maintaining equipment; cleaning up; contributing to logistical tasks including note taking, scheduling, sending email reminders and booking rooms’ (Liboiron et al., 2017, p. 6).^5^ To the list may be added contributions of technicians and programmers.

Such considerations are a reminder that what counts as a significant contribution might not be something once and for all given. They should also remind us to keep an open mind to:

- 6. Apply a wide conception of contributor, including considering whether a set of contributors should be credited as a group.

When it comes to listing contributions by individuals participating in CS in contributor lists, there is limited reason to hold back. For any worthwhile contribution facilitating the specific research project, it is adequate to list it, for the sake of transparency and fairness, unless the contributor disagrees. It may in principle include drivers making considerable contributions transporting equipment or mechanics/technicians maintaining equipment. And as discussed at some length above, crediting a group with authorship may be adequate for the same reasons.

Finally, we propose the application of a meta-rule in the form of benefit of the doubt:

- 7. When in doubt, err on the side of generosity towards CS participants.

As a consequence of the fluctuating conceptions of credit and contributorship, there are likely to be some borderline cases regarding who should be included as co-author. In such cases, we suggest that one should opt for erring on the side of generosity; that is, it is preferable that someone *almost* deserving is included to someone barely deserving being excluded. Including those not deserving and excluding those deserving being credited authorship is equally bad when it comes to desert, honesty, transparency, and responsibility. However, we suggest that generosity at the margin has positive dynamic effects since it encourages engagement among contributors to a project. It would also increase the chance of CS participants being rightfully credited. It should, of course, be considered in the individual case whether the most adequate crediting for contributions take the form of authorship or contributorship.

Admittedly, there might be some risks of over-inclusion of co-authors. For instance, imagine a group of CS participants who possess valuable data (for instance, a long time series of observations of animal behaviour in some specific area) but who refuse to contribute the data to a project unless they are listed as authors. This is a situation not unlike ones where hostage authorship can occur.

Despite the risk of over-inclusion, the heuristic is sound. First, the problem is likely not a huge one. It applies only as a tiebreaker in borderline cases. In the majority of situations, the question of whom to include as author is not as difficult. Second, and more importantly, in situations where authorship is at stake, the power relationship between CS participants and professional researchers is not an equal one. The professionals are typically in a superior position. There is a long history of marginalization of some authors, for instance based on their gender identity (Hassoun et al., 2022; Heath-Stout, 2020; Ross et al., 2022). There are good reasons to promote equity in authorship, and therefore erring on the side of generosity is warranted—arguably more so in CS than in professional science.

## Conclusion

We have argued that considerations of desert, transparency and fairness speak in favor of including CS participants as authors, and that this is compatible with the Vancouver guidelines whenever CS participants do fulfil the authorship criteria. Much criticism against these authorship criteria from a CS perspective seems to concern unfair and non-transparent authorship practices rather than the very guidelines. Thus, we do not propose radical reform of existing authorship guidelines. It is possible, however, that future developments might require such reform, for example in relation to group contributions. We propose seven heuristic rules to help dealing with authorship issues in citizen science: (1) As far as possible, respect existing guidelines (such as ICMJE’s Vancouver guidelines), (2) Ensure that no authors who fulfil the criteria of the Vancouver guidelines are left out, (3) Use existing resources for specifying roles of contributors, (4) Look to nearby fields, (5) Apply a wide conception of contributions, (6) Apply a wide conception of contributor, including considering whether a set of contributors should be credited as a group, and (7) When in doubt, err on the side of generosity towards CS participants.

## Footnotes

1 For a terminological overview, see Haklay et al. (2021). A common distinction is the following: On the one hand, there is CS that is initiated and run by professionals, typically academics, with volunteer participants providing data from observations. This is sometimes referred to as ‘contributory CS’ (Cooper & Lewenstein, 2016) or ‘professional-driven inquiry’ (Resnik, 2019a). On the other hand, there is ‘democratized CS’ (Cooper & Lewenstein, 2016), or ‘citizen-driven inquiry’ where the research is “initiated, designed, and conducted by citizens” (Resnik, 2019a, p. 21). A list of terms related by way of family resemblance to ‘citizen science’ can be found in Pelacho et al. (2021).

2 For an overview of different forms of recognition in CS, see Sandin & Baard (2024).

3 The use of generative AI tools in research involves many serious ethical considerations in addition to the question of whether such tools might be listed as authors. Those are beyond the scope of the present paper.

4 This problem is not unique to CS. Similar considerations might apply in activities like participatory research. Consider for instance the study’Coping with Covid in Prison: The Impact of the Prisoner Lockdown’ (User Voice, 2022), in which both ex-prisoners and current prisoners participated as co-researchers. In this case, it might not be favorable for those involved to be listed as co-authors given the stigma associated with having been in prison. To the contrary, there might be good reasons—ethically speaking—not to list them even if they have made a sufficiently substantial contribution to the study and fulfil the Vancouver guidelines.

5 Liboiron et al. are in fact even more radical, arguing that also ‘caring for members’ physical and mental health by listening, sending sick people home, providing “time outs,” and telling jokes; and thanking each other’ (Liboiron et al., 2017, pp. 6–7) should be taken into consideration when considering the ordering of authors, that is, whether one is to be given a more or less prominent position in the author list of a published article (though not for awarding authorship in the first place).
